# Profile and Content of Residual Alkaloids in Ten Ecotypes of *Lupinus mutabilis* Sweet after Aqueous Debittering Process

**DOI:** 10.1007/s11130-020-00799-y

**Published:** 2020-02-03

**Authors:** Paola Cortés-Avendaño, Marko Tarvainen, Jukka-Pekka Suomela, Patricia Glorio-Paulet, Baoru Yang, Ritva Repo-Carrasco-Valencia

**Affiliations:** 1 Universidad Nacional Agraria La Molina-UNALM, Av. La Molina s/n, Lima, Peru; 2 Food Chemistry and Food Development, Department of Biochemistry, University of Turku, FI-20014 Turku, Finland

**Keywords:** Gas chromatography, Peruvian lupin seeds, Mass spectrometry, Aqueous debittering process, Toxicity

## Abstract

The evaluation of the level of alkaloids in edible *Lupinus* species is crucial from a food safety point of view. Debittering of lupin seeds has a long history; however, the control of the level of alkaloids after processing the seeds is typically only evaluated by changes in the bitter taste. The aim of this study was to evaluate the profile and residual levels of quinolizidine alkaloids (QA) in (*Lupinus mutabilis* Sweet) after aqueous debittering process. Samples from 10 ecotypes from different areas of Peru were analyzed before and after the process. Based on results obtained by gas chromatography and mass spectrometry, from eight alkaloids identified before the debittering process, only small amounts of lupanine (avg. 0.0012 g/100 g DM) and sparteine (avg. 0.0014 g/100 g DM) remained in the seeds after the debittering process, and no other alkaloids were identified. The aqueous debittering process reduced the content of alkaloids to levels far below the maximal level allowed by international regulations (≤ 0.2 g/kg DM).

**Electronic supplementary material:**

The online version of this article (10.1007/s11130-020-00799-y) contains supplementary material, which is available to authorized users.

## Introduction

The genus *Lupinus* is a member of the legume family Fabaceae. Oceania and Eurasia contribute over 90% of the 1.3 Mt annual world lupin production followed by the countries of Africa (5–7%) and South, North, and Central America (3–5%) [1, 2]. The edible lupin species with the highest alkaloid content is *L. mutabilis*, which originates from South America [3, 4]. It is grown at altitudes between 2000 and 3850 m above sea level at the high Andean areas (mainly Ecuador, Bolivia, and Peru), and also in Colombia and Argentina [5].

Lupin has been recognized as a highly nutritious grain providing relatively high quantity of proteins compared to traditional legumes, as well as high content of essential fatty acids and dietary fiber [5, 6]. Besides nutrients, lupin seeds contain secondary metabolites such as polyphenols, carotenoids, alkaloids and phytosterols with potential antimutagenic, anticarcinogenic, and hypocholesterolemic activities [7–10]. The seeds are often consumed after cooking as whole seeds, but they can also be used as food ingredients (flour) in the production of bread, gluten-free cakes or dairy products [11]. However, a limiting factor for wider use of *L. mutabilis* has been the high content of alkaloids that confers bitter taste to food products and may have acute anticholinergic toxicity, characterized by symptoms such as headache, nervousness, nausea and relaxation of the nictitating membrane of the eye [12]. Preliminary studies on its toxicity suggest acute-lethal dose as present in lupin seeds is 10 mg/kg body weight (b.w.) for infants and 25 mg/kg b.w. in adults [6].

One way for reducing the concentration of alkaloids in *L. mutabilis* is microbial debittering, which involves fermentation with Gram-negative bacterial strains isolated from the soil where lupin has been cultivated [13]. Likewise, debittering processes using different solvents have been tested, but using water for the elimination of alkaloids is the best option in order to reduce solvent waste and hence environmental burden. Debittering of lupin seeds using water has been used since pre-Inca times in the Andean Region [14]. The residual levels of alkaloids have traditionally been verified based on presence or absence of bitter taste only.

Peru is a major producer of lupin in the South America, with a yield exceeding 1 million tonnes in 2017 [15]. Nevertheless, data on the profile of alkaloids in Peruvian lupin ecotypes is very limited. Hatzold et al. [16] investigated a wide range of ecotypes of lupin from the south of Peru; identifying different alkaloids (13-hydroxylupanine, 17-oxosparteine and 11, 12-dehydrosparteine), however, ecotypes from the northern and central of Peru were not included despite the great nutritional and commercial importance of lupin in these regions. Gross et al. [17] measured the alkaloid content of low-alkaloid lupin *L. mutabilis* 0.0075% variety “Inti” and 0.015% in variety “2150” cultivars from Chile. The investigation did not include more bitter ecotypes and took no notice of varietal differences, as the study was based on commercial products. Information regarding alkaloid content and profile in some bitter seeds of *L. mutabilis* is available, yet the literature is devoid of information concerning the alkaloid content and profile in different ecotypes after the aqueous debittering process of seeds. The knowledge of the alkaloid profile of debittered lupin will allow evaluation of the toxicity and bioactivities of alkaloids that remain in processed seeds. Therefore, the aim of this study was to evaluate the effect of aqueous debittering process on the profile and content of alkaloids in different ecotypes of *L. mutabilis* by using chromatographic and mass spectrometric techniques.

## Materials and Methods

### Plant Material

Ten ecotypes of bitter lupin (*Lupinus mutabilis*) were chosen according to the geographical distribution, production area and commercial interest. When the pods turned yellow and reached harvest maturity, the seeds were harvested by hand by local producer and collected by Program of Legumes of National Agraria La Molina University of Lima Peru. The seeds were placed in paper bags and stored at 13–14% relative humidity at 20 °C. The seeds originate from different Regions of the south, center, and north of Peru (pictures shown in Supplementary Material 1): (E1) Cajamarca; (E2) Altagracia from Huamachuco-La Libertad; (E3) Paton grande from Otuzco-La Libertad; (E4) Cholo fuerte from Ancash; (E5) Huanuco I from Santa Rosa-Marambuco-Huanuco; (E6) Compuesto blanco semi precoz INIA from Santa Ana- Huancayo-Junin; (E7) H6 INIA from Junin; (E8) Moteado beige from Jauja-Junin; (E9) Andenes INIA from Cusco; (E10) Yunguyo from Puno.

### Chemicals

Sparteine (97%), angustifoline (98%), α-isolupanine perchlorate (>95%) and caffeine (≥ 95%) were purchased from ChemFaces (Wuhan, Hubei, China). (+)-lupanine perchlorate (97%) were purchased from Santa Cruz Biotechnology, Inc. (Dallas, TX, USA). Trichloroacetic acid was purchased from Sigma-Aldrich Co. (St. Louis, MO, USA). Hydrochloric acid (reagent grade, 37%), sodium sulfate (anhydrous, granular, ≥ 99%), and HPLC grade methanol, acetone, acetonitrile, dichloromethane, and *n*-hexane were purchased from VWR (Espoo, Finland), and the *n*-alkane series (C8-C40) from AccuStandard, Inc. (New Haven, CT, USA). Sodium hydroxide (technical grade) was purchased from Merck Group (Darmstadt, Germany). Water was purified with the Elga Purelab Ultra water purification system (Elga LabWater, Woodridge, Il, USA) equipped with a 0.2 μm particle filter.

### Aqueous Debittering Process

The seeds were debittered according to the method by Jacobsen and Mujica [5] with slight modifications. Seeds were selected and soaked with seed-to-water ratio 1:6 (*w*/*v*) for 12 h at room temperature. Then the seeds were cooked for 1 h at atmospheric pressure at 241 m above sea level. During the cooking, the water was changed once after 30 min. Following cooking, the seeds were washed with running water for 5 days. After washing the seeds were dried at 50 °C for 18 h. Both seeds (bitter and debittered) were milled in a hammer mill, to obtain particles between 100 and 500 μm. The flours were packed in polyethylene bags.

### Extraction of Alkaloids

Alkaloid extraction was carried out according to the method of Muzquiz et al. [18] with some modifications. Finely ground lupin seeds were weighted accurately (c.a 0.1 g bitter and 1 g debittered) and caffeine (100 μg) was added as the internal standard. The seeds were then homogenized three times each with 5 mL of 5% trichloroacetic acid using a T25 digital Ultra-Turrax high-performance disperser (IKA Werke GmbH & Co. KG, Staufen, Germany) at a speed of 10.6 rpm for 1 min, followed by centrifugation (10,000 x *g*) for 10 min at room temperature. The supernatants of the three extractions were combined and hydrolyzed with 0.8 mL of 10 M NaOH, and the alkaloids were extracted three times, each with 15 mL of dichloromethane. The dichloromethane extracts were combined and evaporated to dryness with a rotary evaporator at 30 °C. The residue was dissolved in 1 mL of methanol and filtered through a 0.22 μm PTFE membrane filter into a glass vial for analyses.

### Alkaloid Profile Analysis by GC-MS

The analysis was performed following a previously established method by Przybylak et al. [7] with slight modifications. The sample extracts were analyzed using an HP 6890 gas chromatograph couple with an HP 5973 MSD mass detector). An Agilent DB-1MS capillary column was used (30 m × 0.25 mm i.d., 0.25 μm film thickness, Agilent Technologies Inc., Santa Clara, CA, USA). Helium was used as the carrier gas with a flow rate of 1.2 mL/min corresponding to a constant linear velocity of 42 cm/s. The injector temperature was set at 290 °C; the detector at 300 °C. The injection was performed in a split mode, with a ratio of 1/20. The oven temperature program was isothermal at 180 °C for 2 min, then increased to 300 °C at a rate of 6 °C/min, and kept at 300 °C for 10 min. The injection volume was 1 μL. The alkaloids compounds were identified by comparison of their mass spectra with those from pure standards analyzed under the same conditions, and with data of the literature [19] as well as with Wiley 275 MS library. In addition, the retention indices (RI) were experimentally calculated using the homologous series of *n*-alkanes, and were compared to the values with those reported in the literature for GC columns with 5%-phenyl-95%-dimethylpolysiloxane c, and finally spiking with commercial standard compounds.

### Alkaloid Quantification by GC-FID

Quantitative analysis of the alkaloids was performed using a Shimadzu GC-2010 plus GC equipped with flame ionization detector (Shimadzu Corp) and an Agilent DB-1MS column (30 m × 0.25 mm i.d., 0.25 μm film). The injector and detector temperatures were set at 290 and 300 °C, respectively. Elution conditions used were the same described above for GC-MS analysis. Each extract was injected (1uL) in triplicate. Caffeine was used as the internal standard. Peak areas of alkaloids were corrected by applying appropriate relative response factors. The quantification of each alkaloid present in the extracts was achieved from the standard curve of the respective standard analyzed under the same conditions. Since standards of some identified compounds were not commercially available or their degree of purity was not adequate for quantitative purposes, alkaloid quantification was achieved as follows: sparteine, lupanine, angustifoline, and *α*-isolupanine were quantified as themselves using reference compounds whereas nutalline, multiflorine, oxylupanine and 11, 12-dehydrolupanine were quantified as lupanine equivalents.

### Statistical Analysis

A one-way analysis of the variance of the alkaloid content was performed to evaluate the differences between ecotypes. When the ANOVA indicated a significant treatment effect, Tukey’s HSD test was done to determine which treatment means differed significantly. Statgraphics 18 software (Statistical Graphics, Washington, DC, USA) was used.

## Results and Discussion

### Alkaloid Profile in Bitter Seeds

Alkaloids were identified by GC-MS based on their characteristic ions as well as by retention times of standards. Eight alkaloids, sparteine, angustifoline, *α*-isolupanine, lupanine, nutalline, multiflorine, oxylupanine and 11, 12-dehydrolupanine (Table 1; Supplementary Material 2a) were identified. The obtained profile for the lupin ecotypes was generally in agreement with the findings of earlier studies [18, 19]. Although 13-hydroxylupanine, 17-oxosparteine and 11, 12-dehydrosparteine have been described in *L. mutabilis* [16, 17], they were not identified in the ecotypes investigated in our research. Some of the alkaloids detected are common in other species too, *e.g*., angustifoline, lupanine and sparteine in *L. albus* seeds, sparteine in *L. luteus*, lupanine in *L. hispanicus*, and angustifoline in *L. angustifolius* [23–27]. It is worth noting that alkaloids such as *α*-piridone, cytisine, and anagyrine, which are highly poisonous for invertebrates and common in wild species [23], were not found in the samples of *L. mutabilis*. In bitter seeds (Table 2), lupanine was the main alkaloid with a concentration between 2.5–5.2 g/100 DM, constituting on average 77.2% of the total alkaloids. The result is in agreement with the findings by Gross et al. [17] which indicate lupanine to be the main alkaloid in *L. mutabilis*, accounting for more than 80% of total alkaloids. On the other hand, Rybiński et al. [28] found that lupanine in *L. albus* represents 71.3% of the total alkaloids, this value being lower compared to the level in *L. mutabilis*. The concentration of lupanine in this study was higher than the content reported for seeds of *L. mutabilis* from Ecuador [29]. Among the ecotypes studied, E8 (Moteado beige from Junin) had the highest content of lupanine as well as the highest total content of alkaloids. The second most abundant alkaloid was sparteine with a concentration range of 0.2–0.9 g/100 DM. These results are in agreement with the earlier research on the composition of alkaloid in *L. mutabilis* seeds [16]. Sparteine represented on average 9.9% of the total alkaloids, and the ecotype with the highest sparteine content was E5 (Huánuco I). The alkaloids present in smaller proportions are indicated in Table 2. These compounds include angustifoline and multiflorine, both present at an average level of 0.1% of the total alkaloids. The relative proportions of these two alkaloids is lower in comparison with those earlier reported for *L. albus* (angustifoline 3.8% and multiflorine 6.8% of the total alkaloids) [28]. The others alkaloids present in the 10 bitter ecotypes include *α*-isolupanine, nutalline, oxylupanine and 11, 12-dehydrolupanine, representing on average 0.4, 6.8, 4, 1.4% of total alkaloids, respectively. The values of lipophilicity parameter Log P of individual alkaloids are presented in Table 1, showing potential relationships between structure and activity (SAR). Sparteine has the highest Log P (2.5) followed by lupanine, *α*-isolupanine, 11, 12-dehydrolupanine, multiflorine, and angustifoline with an average value of Log P (1.5). The alkaloids with the lowest values of Log P were nutalline and oxylupanine. The alkaloids with high Log P are more hydrophobic, a property facilitating the entry of the alkaloids to the cell through the hydrophobic cell membranes [30]. It is important to pay attention to both the concentration and the structural characteristics of the alkaloids in lupin seeds.

**Table 1:** Alkaloid profile of the samples (GC-MS) and lipophilicity parameter (Log P) of the alkaloids

Peak	Alkaloids	Identification ions	M^+^	ID	RI /Exp	RI/Lit	Log P
1	Caffeine (internal standard)	194/109/82/67/55	194	S	–	–	–
2	Sparteine	98/137/193/234	234	S	1803	1785	2.5
3	Angustifoline	55/94/112/150/193	234	S	2093	2083	1.4
4	*α*- Isolupanine	55/98/136/149/219/248	248	S	2122	2105	1.6
5	Lupanine	55/98/136/149/248	248	S	2176	2165	1.6
6	Nutalline	98/136/150/247/264	264	T	2271	2255	0.6
7	Multiflorine	55/110/134/149/246	246	T	2328	2310	1.5
8	Oxylupanine	55/134/152/165/246/264	264	T	2423	2410	0.6
9	11,12- Dehydrolupanine	134/148/231/246	246	T	2573	2190	1.5

M^+^, molecular ion; RI/Exp, retention indices obtained in this experiment, calculation according to Kovats [20]: *100 (tc-tn/ (tn + 1)-tn) + n*, where *tc* is the retention time of the compound, *tn* is the retention time of the preceding *n- alkane*, *tn + 1* is the retention time of the following *n-*alkane and *n* is the preceding *n-*alkane; RI/Lit: retention indices described in the literature by Wink et al. [19]; ID, identification: S, identified by comparison with an authentic standard, T, tentatively identified by comparison of mass spectrum and RI in literature [19] and NIST 05 library Database [21]. Log P: value of lipophilicity parameter, computed by XLogP3 3.0 [22]

**Table 2:** Alkaloids in bitter and debittered lupin seeds measured by GC- FID

		Individual alkaloids							
Seeds	Ecotypes	Sparteine	Angustifoline	*α*- Isolupanine	Lupanine	Nutalline	Multiflorine	Oxylupanine	11,12- Dehydrolupanine
Bitter	E1	0.640 ± 0.008 ^f^	0.005 ± 0.000 ^bc^	0.015 ± 0.000 ^c^	2.958 ± 0.008 ^b^	0.452 ± 0.006 ^f^	0.012 ± 0.000 ^e^	0.177 ± 0.006 ^bc^	0.083 ± 0.003 ^d^
	E2	0.421 ± 0.005 ^d^	0.004 ± 0.000 ^a,b^	0.021 ± 0.000 ^d^	4.307 ± 0.146 ^d^	0.505 ± 0.008 ^g^	0.003 ± 0.000 ^b^	0.141 ± 0.011 ^ab^	0.052 ± 0.000 ^b^
	E3	0.592 ± 0.017 ^e^	0.013 ± 0.000 ^g^	0.012 ± 0.001 ^ab^	4.262 ± 0.179 ^d^	0.546 ± 0.015 ^h^	0.008 ± 0.000 ^d^	0.220 ± 0.014 ^cd^	0.077 ± 0.003 ^cd^
	E4	0.774 ± 0.029 ^g^	0.003 ± 0.000 ^a^	0.014 ± 0.000 ^bc^	3.322 ± 0.014 ^c^	0.375 ± 0.030 ^e^	0.010 ± 0.000 ^d^	0.099 ± 0.001 ^a^	0.031 ± 0.000 ^a^
	E5	0.890 ± 0.017 ^h^	0.007 ± 0.000 ^e^	0.010 ± 0.000 ^a^	2.504 ± 0.044 ^a^	0.281 ± 0.008 ^cd^	0.006 ± 0.000 ^c^	0.160 ± 0.008^b^	0.047 ± 0.001 ^ab^
	E6	0.274 ± 0.004 ^b^	0.009 ± 0.000 ^f^	0.027 ± 0.000 ^e^	4.164 ± 0.026 ^d^	0.314 ± 0.004 ^d^	0.001 ± 0.000 ^a^	0.263 ± 0.016 ^de^	0.099 ± 0.003 ^e^
	E7	0.423 ± 0.008 ^d^	0.009 ± 0.000 ^f^	0.019 ± 0.000 ^d^	3.354 ± 0.061 ^c^	0.262 ± 0.008 ^bc^	0.003 ± 0.000 ^b^	0.233 ± 0.013 ^cd^	0.062 ± 0.002 ^bc^
	E8	0.196 ± 0.002 ^a^	0.009 ± 0.000 ^ef^	0.027 ± 0.000 ^e^	5.231 ± 0.045 ^f^	0.145 ± 0.003 ^a^	0.001 ± 0.001 ^a^	0.294 ± 0.016 ^e^	0.073 ± 0.003 ^cd^
	E9	0.377 ± 0.003 ^c^	0.009 ± 0.000 ^f^	0.020 ± 0.000 ^d^	4.771 ± 0.065 ^e^	0.236 ± 0.010 ^b^	0.001 ± 0.000 ^a^	0.266 ± 0.026 ^de^	0.099 ± 0.001 ^e^
	E10	0.367 ± 0.014 ^c^	0.005 ± 0.000 ^d^	0.016 ± 0.002 ^c^	3.612 ± 0.261 ^c^	0.275 ± 0.022 ^bcd^	0.001 ± 0.000 ^a^	0.149 ± 0.047 ^ab^	0.069 ± 0.016 ^cd^
Debittered	E1	0.002 ± 0.000 ^e^	n/d	n/d	0.003 ± 0.000 ^c^	n/d	n/d	n/d	n/d
	E2	0.001 ± 0.000 ^bc^	n/d	n/d	0.001 ± 0.000 ^a^	n/d	n/d	n/d	n/d
	E3	0.001 ± 0.000 ^a^	n/d	n/d	0.001 ± 0.000 ^a^	n/d	n/d	n/d	n/d
	E4	0.002 ± 0.000 ^d^	n/d	n/d	0.001 ± 0.000 ^a^	n/d	n/d	n/d	n/d
	E5	0.001 ± 0.000 ^ab^	n/d	n/d	0.001 ± 0.000 ^a^	n/d	n/d	n/d	n/d
	E6	0.001 ± 0.000 ^bc^	n/d	n/d	0.001 ± 0.000 ^a^	n/d	n/d	n/d	n/d
	E7	0.001 ± 0.000 ^bc^	n/d	n/d	0.001 ± 0.000 ^a^	n/d	n/d	n/d	n/d
	E8	0.001 ± 0.000 ^ab^	n/d	n/d	0.002 ± 0.000 ^b^	n/d	n/d	n/d	n/d
	E9	0.001 ± 0.000 ^c^	n/d	n/d	0.001 ± 0.000 ^a^	n/d	n/d	n/d	n/d
	E10	0.001 ± 0.000 ^ab^	n/d	n/d	0.001 ± 0.000 ^a^	n/d	n/d	n/d	n/d

Data (g/100 g DM) is presented as mean ± SD and represents a mean of minimum of three independent measurements. Values of bitter and debittered seeds with unlike letters (a-h) and (a-e) within the same column differ significantly (*p* < 0.05) respectively. GC-FID, gas chromatography-flame ionization detection; E, ecotype (see Supplementary Material 1); *n/d* not detected

### Alkaloid Profile in Debittered Seeds

After the aqueous debittering process, only two alkaloids, sparteine and lupanine were identified in the 10 ecotypes, while the other alkaloids that were present in lower concentrations in bitter seeds were not detected (Supplementary Material 2b). Table 2 shows that the content of lupanine and sparteine were only 0.001–0.002 and 0.001–0.003 g/100 DM, respectively. After debittering, sparteine represents on average 54% and lupanine 46% of the total alkaloids. The content of lupanine and sparteine, the major alkaloids in *L. mutabilis*, were decreased on average by 99.9 and 99.7%, respectively, by the aqueous debittering process.

The level of the decrease in lupanine content is very similar to that obtained by Santana et al. [13], who reported elimination of 99% of the initial content of lupanine present in *L. albus* seeds after a fermentation process. During fermentation, bacteria used lupanine as a source of carbon and energy. Chilomer et al. [31] observed that after germination the alkaloid content was increased.

As presented in Table 1, The Log P of sparteine is higher than that of lupanine. This feature explains the presence of sparteine in the debittered seeds despite its lower concentration before the debittering process compared with lupanine. The value Log P may be very significant because of the structure-activity relationship. In this regard, Pothier et al. [32] reported that lupanine is less toxic than sparteine; at high dose, sparteine stops the heart in diastole and at low dose it reduces coronary flow, contraction amplitude and heart rate. Lupanine and *α*-isolupanine have the same value of Log P (Table 1); however, only lupanine could be identified in the debittered seeds. This difference can be attributed to the initial concentrations before the debittering and the molecular structure of each alkaloid.

### Alkaloid Content in Bitter Seeds

**Fig. 1:**
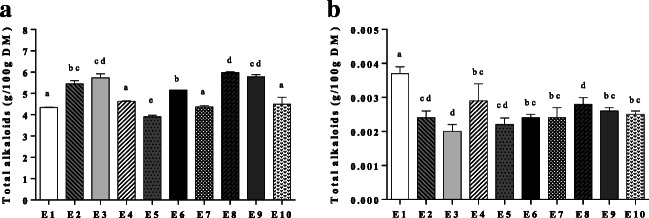
Total alkaloids **a** Before the debittering process; **b** after the debittering process. Bars represent standard deviation of three independent replicates. Values with unlike letters (a-e) differ significantly (*p* < 0.05)

The total content of alkaloids in bitter lupin seeds is shown in Fig. 1a. All the ecotypes can be considered bitter varieties, because they contained alkaloids at a level above 5 g/100 g DM, whereas the contents in non-bitter varieties are generally between 0.01–0.05 g/100 g DM [29]. The ecotypes with the highest content of alkaloids were E2, E3, E6, E8, and E9, each reaching a level close to 6 g/100 g DM. Ecotypes E2 and E3 were from the same region but from different altitudes (3350 and 3496 m from the sea level, respectively), whereas ecotypes E6 and E8 were from the central part of Peru and the ecotype E9 from southern Peru, but they were all cultivated at similar altitudes. Ecotypes containing intermediate levels of alkaloids (almost 5 g/100 g DM) were E1, E4, E7 and E10, and the ecotype containing the least alkaloids was E5 with a total content of 4 g/100 g DM. The ecotypes with the highest alkaloid content were grown at locations over 3280 m above sea level. In these high Andean areas, the climate is dry and temperature conditions are mild. At a low humidity level, alkaloids tend to accumulate [6]. In ecotypes grown at an altitude below 3271 and above 3761 m, the content of alkaloids decreased. Apart from latitude and altitude, differences in total alkaloid content *(e.g*., ecotype E7 compared with E8) may be explained by *e.g*., varietal differences, distribution of alkaloids inside the plant and the soil of the cultivation site [33]. In lupins, the total alkaloid content varies significantly between ecotypes and varieties. In this study, the total alkaloid content of non-processed seeds was 4–6 g/100 g DM, which is higher than those reported in some previous studies of *L. mutabilis*. For example, Hatzold et al. [16] reported a total alkaloid content of 3.1% in *L. mutabilis*, and Gross et al. [17] 0.0075% in low alkaloid *L. mutabilis* variety “Inti” and 0.015% in variety “2150”. The alkaloid contents measured in the present study were also higher compared with high-alkaloid varieties of other lupin species such as *L. campestri, L. angustifolius, L. hispanicus, L. luteus and L. albus* with alkaloid contents varying between 1.9 and 2.7% [29].

### Alkaloid Content in Debittered Seeds

The total content of alkaloids in lupin seeds after the debittering process is shown in Fig. 1b. All the ecotypes had a very low residual alkaloid content with an average of 0.003 g/100 g DM. The debittered seeds had a much lower alkaloid content than the safe limit (20 mg/100 g) indicated for safe human and animal consumption by the health authorities of the UK, France and Australia [34]. The decrease is due to solubilization in water, thermal degradation and the cell wall permeability, facilitating the extraction of alkaloids [23, 33]. There are differences in the percentage of decrease in alkaloids among ecotypes, depending on the grain structure and composition [35]. The lupin seed cover plays an important role at the time of water diffusion, mainly at the hilum level and hilar fissure, which is the main entrance of water to the grain. On the other hand, the surface of the seed coat is covered by a cuticle that provides barrier for permeability. The cuticle contains different types of hydrophobic substances such as wax, lignin polysaccharides, pectin, calluses, quinones, suberin, cutin, and phenols [35]. Previous research data has shown that it is necessary to remove alkaloids with debittering process from bitter lupin seeds due to the high alkaloid content [32]. The results of our study demonstrate that the alkaloid content in seeds of the 10 Peruvian ecotypes of *L. mutabilis* is well above the acceptable limit so that it is necessary to perform the debittering process for this species. Data from debittered seeds demonstrate that the residual alkaloid levels are below safety limits. In the future, isolated alkaloids can be selected for different applications such pharmacological properties and fertilizers. Seeds from the high Andean regions of Junin and Huanuco, which have a high content of total alkaloids with the highest content of lupanine and highest content of sparteine, respectively, could be used for this purpose.

## Conclusion

The effect of the aqueous debittering process on the profile and content of alkaloids was investigated in lupin seeds of different ecotypes cultivated in diverse regions in Peru. In the ten ecotypes studied, eight quinolizidine alkaloids were identified, of which lupanine and sparteine were the major compounds. The content of alkaloids was influenced by geographical location likely due to the different climatic conditions. The aqueous water debittering process reduced the level of alkaloids to levels far below the maximal level allowed by international regulations. The control of the level of alkaloids is essential in the prevention of potential food safety problems. The utilized GC-MS and GC-FID methods proved to be suitable for the identification and quantification of alkaloids in lupin. This is the first report on the alkaloid profile and content of different ecotypes of Peruvian lupin before and after debittering process, hence giving a possibility to relate quantitative and qualitative results provided by modern analytics to the aqueous debittering technique, both contributing to food safety.

## Electronic supplementary material

ESM 1 (PDF 244 kb)

ESM 2 (PDF 370 kb)

## Abbreviations

E: Ecotype
DM: Dry matter
RT: Retention time
SD: Standard deviation

## References

[1] Lucas MM, Stoddard FL, Annicchiarico P, Frías J, Martínez-Villaluenga C, Sussmann D, Duranti M, Seger A, Zander PM, Pueyo JJ (2015). The future of lupin as a protein crop in Europe. Front Plant Sci.

[2] Vargas-Guerrero P, García-López M, Martínez-Ayala AL, Domínguez-Rosales JA, Gurrola-Díaz CM (2014) Administration of *Lupinus albus* gamma conglutin (Cγ) to n5 STZ rats augmented Ins-1 gene expression and pancreatic insulin content. Plant Foods Hum Nutr 69:241–247. 10.1007/s11130-014-0424-y 10.1007/s11130-014-0424-y 24894193

[3] Hatzold T, Elmadfa I, Gross R (1983). Edible oil and protein concentrate from *Lupinus mutabilis*. Plant Foods Hum Nutr.

[4] Santos C, Ferreira R, Teixeira A (1997). Seed proteins of *Lupinus mutabilis*. J Agric Food Chem.

[5] Jacobsen S-EM, Mujica A (2006) El tarwi (*Lupinus mutabilis sweet*) y sus parientes silvestres, in Botánica Económica de los Andes Centrales. Bot Econ Andes Cent 28:458–482

[6] Carvajal-Larenas FE, Linnemann AR, Nout MJR, Koziol M, van Boekel M (2016) *Lupinus mutabilis*: composition, uses, toxicology, and debittering. Crit Rev Food Sci Nutr 56:1454–1487. 10.1080/10408398.2013.772089 10.1080/10408398.2013.772089 26054557

[7] Przybylak JK, Ciesiołka D, Wysocka W (2005). Alkaloid profiles of Mexican wild lupin and an effect of alkaloid preparation from *Lupinus exaltatus* seeds on growth and yield of paprika (*Capsicum annuum* L). Ind Crops Prod.

[8] Ranilla LG, Apostolidis E, Genovese MI (2009). Evaluation of indigenous grains from the Peruvian Andean region for antidiabetes and antihypertension potential using *in vitro* methods. J Med Food.

[9] Carvajal-Larenas FE, Van Boekel MJAS, Koziol M (2014). Effect of processing on the diffusion of alkaloids and quality of *Lupinus mutabilis* sweet. J Food Process Preserv.

[10] Ruiz-López MA, Barrientos-Ramírez L, García-López PM et al (2019) Nutritional and bioactive compounds in Mexican lupin beans species: a mini-review. Nutrients 11:1785. 10.3390/nu11081785 10.3390/nu11081785 PMC6723436 31382375

[11] Villarino CBJ, Jayasena V, Coorey R et al (2017) Nutritional, health, and technological functionality of lupin flour addition to bread and other baked products: benefits and challenges. Crit Rev Food Sci Nutr 56(5):835–857. 10.1080/10408398.2013.814044 10.1080/10408398.2013.814044 25675266

[12] Khan MK, Karnpanit W, Nasar-Abbas SM (2015). Phytochemical composition and bioactivities of lupin: a review. Int J Food Sci Technol.

[13] Santana F, Fialho A, Sfi-Correia I, Empis J (1996). Isolation of bacterial strains capable of using lupanine, the predominant quinolizidine alkaloid in white lupin, as sole carbon and energy source. J Ind Microbiol.

[14] Torres F, Nagata A, Dreifuss W (1980). Methods of eliminating alkaloids from the seeds of *Lupinus mutabilis* sweet. Arch Latinoam Nutr.

[15] FAOSTAT http://www.fao.org/faostat/en/?#data/QC. Accessed 24 Jun 2019

[16] Hatzold T, Elmadfa I, Gross R et al (1983) Quinolizidine alkaloids in seeds of *Lupinus mutabilis*. J Agric Food Chem 31:934–938. 10.1021/jf00119a003

[17] Gross R, von Baer E, Koch F et al (1988) Chemical composition of a new variety of the Andean lupin (*Lupinus mutabilis* cv. Inti) with low-alkaloid content. J Food Compos Anal 1:353–361. 10.1016/0889-1575(88)90035-X

[18] Muzquiz M, Cuadrado C, Ayet G (1994). Variation of alkaloid components of lupin seeds in 49 genotypes of *Lupinus albus* from different countries and locations. J Agric Food Chem.

[19] Wink M, Meibner C, Witte L (1995) Patterns of quinolizidine alkaloids in 56 species of the genus *Lupinus*. Phytochemistry 38:139–153. 10.1016/0031-9422(95)91890-D

[20] Kovats E (1958) Gaz-chromatographis che charakterisierung organis hcher Verbindungen. Teil 1: Retentionsindices aliphatischer Halogenide, Alkohole, Aldehyde und Ketone. Helv Chim Acta 41:1915–1932. 10.1002/hlca.19580410703

[21] WebBook (2016) NIST chemistry WebBook. WebBook, http://webbook.nist.gov/. Accessed 01.10.16

[22] PubChem (2019) https://pubchem.ncbi.nlm.nih.gov. Accessed 01.08.19

[23] Jimenez-Martinez C (2001). Effect of aqueous and alkaline thermal treatments on chemical composition and oligosaccharide, alkaloid and tannin contents of *Lupinus campestris* seeds. J Sci Food Agric.

[24] Aniszewski T (2015) Alkaloid chemistry. Alkaloids:99–193. 10.1016/B978-0-444-59433-4.00002-X

[25] Magalhães SCQ, Fernandes F, Cabrita ARJ et al (2017) Alkaloids in the valorization of European *Lupinus* spp. seeds crop. Ind Crop Prod 95:286–295. 10.1016/j.indcrop.2016.10.033

[26] Musco N, Cutrignelli MI, Calabrò S (2017). Comparison of nutritional and antinutritional traits among different species (*Lupinus albus* L.; *Lupinus luteus* L.; *Lupinus angustifolius* L.) and varieties of lupin seeds. J Anim Physiol Anim Nutr.

[27] Romeo FV, Fabroni S, Ballistreri G, Muccilli S, Spina A, Rapisarda P Characterization and antimicrobial activity of alkaloid extracts from seeds of different genotypes of *Lupinus* spp. Sustainability 10:1–12. 10.3390/su10030788

[28] Rybiński W, Kroc M, Święcicki W, Wilczura P, Kamel K, Barzyk P, Mikulski W, Brazauskas G (2018). Preliminary estimation of variation of alkaloids content in white lupin (*Lupinus albus* L.) collection. Breeding grasses and protein crops in the era of genomics.

[29] Jiménez-Martínez C, Hernández-Sánchez H, Dávila-Ortiz G (2007). Diminution of quinolizidine alkaloids, oligosaccharides and phenolic compounds from two species of Lupinus and soybean seeds by the effect of *Rhizopus oligosporus*. J Sci Food Agric.

[30] Kučerka N, Gallová J, Uhríková D (2019). The membrane structure and function affected by water. Chem Phys Lipids.

[31] Chilomer K, Kasprowicz-Potocka M, Gulewicz P, Frankiewicz A (2013). The influence of lupin seed germination on the chemical composition and standardized ileal digestibility of protein and amino acids in pigs. J Anim Physiol Anim Nutr (Berl).

[32] Pothier J, Galand N, Dormeau C, Viel C (1998) A comparative study of the effects of sparteine, lupanine and lupin extract on the central nervous system of the mouse. J Pharm Pharmacol 50:949–954. 10.1111/j.2042-7158.1998.tb04013.x 10.1111/j.2042-7158.1998.tb04013.x 9751462

[33] Frick KM, Foley RC, Garg G (2018). Characterization of the genetic factors affecting quinolizidine alkaloid biosynthesis and its response to abiotic stress in narrow - leafed lupin (*Lupinus angustifolius* L.). Plant Cell Environ.

[34] Calabrò S, Cutrignelli MI, Lo Presti V, Tudisco R, Chiofalo V, Grossi M, Infascelli F, Chiofalo B (2015). Characterization and effect of year of harvest on the nutritional properties of three varieties of white lupine (*Lupinus albus* L.). J Sci Food Agric.

[35] Miano AC, García JA, Augusto PED (2015). Correlation between morphology, hydration kinetics and mathematical models on Andean lupin (*Lupinus mutabilis* sweet) grains. LWT-Food Sci Technol.

