# Supplementary material for: Profile and Content of Residual Alkaloids in Ten Ecotypes of Lupinus mutabilis Sweet after Aqueous Debittering Process
Source: Plant Foods Hum Nutr. 2020 Feb 3;75(2):184–91. doi: 10.1007/s11130-020-00799-y (PMC7266797; doi:10.1007/s11130-020-00799-y)
Supplement: Supplementary file 1 — (PDF 244 kb) [file 11130_2020_799_MOESM1_ESM.pdf]

Plant Foods for Human Nutrition. **Profile and content of residual alkaloids in ten ecotypes of *Lupinus mutabilis* Sweet after aqueous debittering process.** Paola Cortés-Avendaño, Marko Tarvainen, Jukka-Pekka Suomela,*, Patricia Glorio-Paulet, Baoru Yang, and Ritva Repo-Carrasco-Valencia.

*Food Chemistry and Food Development, Department of Biochemistry, University of Turku, e-mail: jusuom@utu.fi

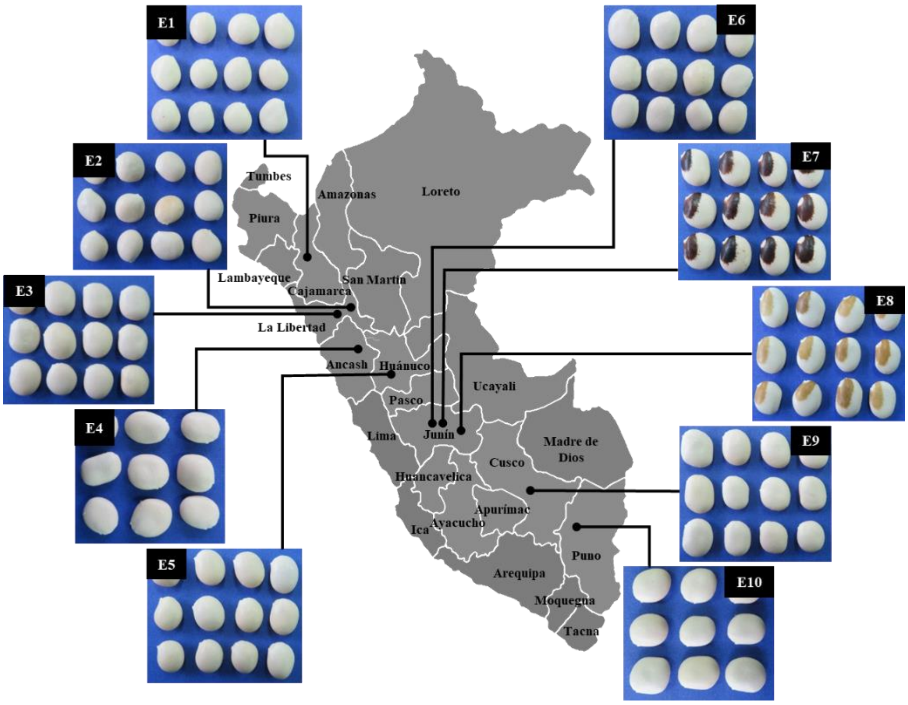

Fig. S1. Different ecotypes of lupins (*Lupinus mutabilis* Sweet) in Peru: (E1) Cajamarca (latitude 7°9'S, longitude 78°26′O, 2780 m above sea level); (E2) Altagracia from Huamachuco-La Libertad (latitude 7°34′S, longitude 78°3′O, 3350 m above sea level); (E3) Paton grande from Otuzco-La Libertad (latitude 7°54'S, longitude 78°33'O, 3496 m above sea level); (E4) Cholo fuerte from Ancash (latitude 9°20′S, latitude 77°10′O, 3150 m above sea level); (E5) Huanuco I from Santa Rosa-Marambuco-Huanuco (latitude 9°56'S, longitude 76°12'O, 3761 m above sea level); (E6) Compuesto blanco semi precoz INIA from Santa Ana- Huancayo-Junin (latitude 12°00'S, longitude 75°13'O, 3295 m above sea level); (E7) H6 INIA from Junin (latitude 12°4′S, longitude 75°13′O, 3271 m above sea level; (E8) Moteado beige from Jauja-Junin (latitude 12°01'S, longitude

75°14'O, 3316 m above sea level); (E9) Andenes INIA from Cusco (latitude 13°31′S, longitude 71°58′O, 3400 m above sea level); (E10) Yunguyo from Puno (latitude 16°15′S, longitude 69°05′O, 3800 m above sea level).
