# Supplementary material for: Profile and Content of Residual Alkaloids in Ten Ecotypes of Lupinus mutabilis Sweet after Aqueous Debittering Process
Source: Plant Foods Hum Nutr. 2020 Feb 3;75(2):184–91. doi: 10.1007/s11130-020-00799-y (PMC7266797; doi:10.1007/s11130-020-00799-y)
Supplement: Supplementary file 2 — (PDF 370 kb) [file 11130_2020_799_MOESM2_ESM.pdf]

Plant Foods for Human Nutrition. **Profile and content of residual alkaloids in ten ecotypes of *Lupinus mutabilis* Sweet after aqueous debittering process.** Paola Cortés-Avendaño, Marko Tarvainen, Jukka-Pekka Suomela,*, Patricia Glorio-Paulet, Baoru Yang, and Ritva Repo-Carrasco-Valencia.

*Food Chemistry and Food Development, Department of Biochemistry, University of Turku, e-mail: jusuom@utu.fi

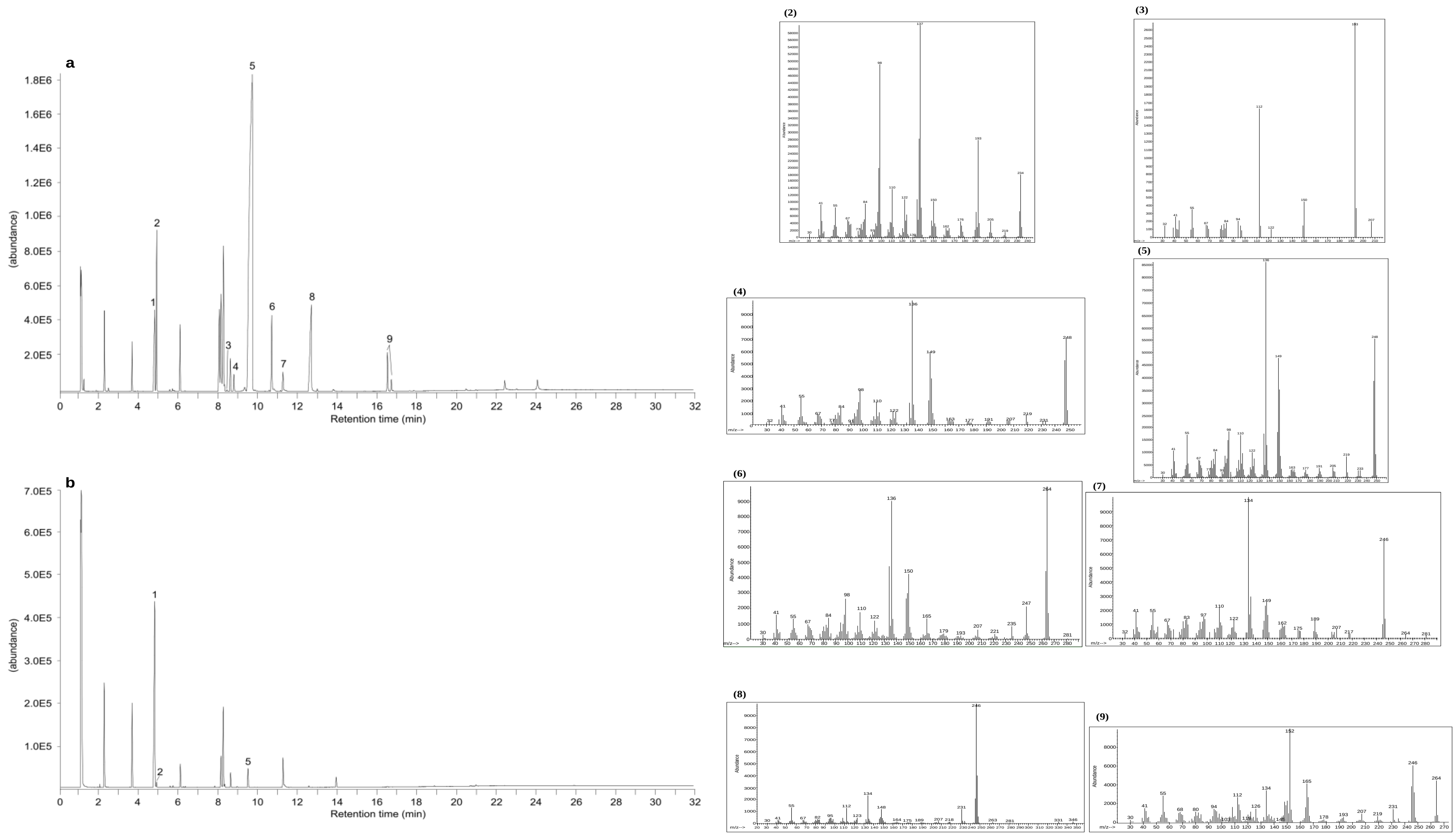

Fig. S2. GC-MS chromatographic profiles and spectra of alkaloids in the ecotype E8, before and after the aqueous debittering process (**a** and **b,** respectively): (**1**) caffeine (internal standard); (**2**) sparteine; (**3**) angustifoline; (**4**) α-isolupanine; (**5**) lupanine; (**6**) nuttaline; (**7**) multiflorine; (**8**) oxylupanine; (**9**) 11, 12-dehydrolupanine.
